# Whole-Genome Sequencing of SARS-CoV-2 Strains from Asymptomatic Individuals in India

**DOI:** 10.1128/MRA.00850-21

**Published:** 2022-01-27

**Authors:** Ambica Rangaiah, Sathyanarayan Muthur Shankar, Shashiraja Padukone, Kusuma Gowdra Rangappa, Shantala Gowdara Basawarajappa

**Affiliations:** a Department of Microbiology, Bangalore Medical College Research Institute, Bengaluru, Karnataka, India; KU Leuven

## Abstract

We performed targeted genome sequencing of 11 severe acute respiratory syndrome coronavirus 2 (SARS-CoV-2) samples collected from asymptomatic individuals. These nasopharyngeal and oropharyngeal samples were collected during the first wave of coronavirus disease 2019 (COVID-19) in Karnataka, India. Nine strains were found to be Nextstrain clade 20B (PANGO lineage B.1.1.519, GISAID clade GR), and two were identified as clade 20A (PANGO lineage B.1.619, GISAID clade G). The spike protein mutation D614G was observed across all the sequenced strains.

## ANNOUNCEMENT

In December 2019, severe acute respiratory syndrome coronavirus 2 (SARS-CoV-2) was first detected in China (1). After that, it rapidly spread across the globe. On 11 March 2020, the World Health Organization declared coronavirus disease (COVID-19) to be a pandemic (2). SARS-CoV-2 belongs to the genus *Betacoronavirus* and family *Coronaviridae* and contains a positive-sense, single-stranded RNA genome of approximately 30 kb. India confirmed its first COVID-19 case on 30 January 2020 (3). As of September 2021, India was preparing for the third wave of COVID-19. On 10 August 2021, India recorded 31,969,954 confirmed cases with 428,300 deaths, which was the second-highest COVID-19 burden worldwide, following the United States (4). In India, as of 26 May 2021, the state of Karnataka had recorded the second-highest number of COVID-19 cases, after Maharashtra State (5). We herein present the whole-genome data of SARS-CoV-2 strains obtained from asymptomatic individuals, collected during the first wave of COVID-19 (August 2020 to December 2020).

This study was approved by the ethics committee of Bangalore Medical College and Research Institute (number BMCRI/PS/09/2020-21). The nasopharyngeal and oropharyngeal swabs, which had been collected in a viral transport medium from asymptomatic individuals having had primary contact with COVID-19-positive cases or who had given the sample as a part of a surveillance program, were retrieved from a −80°C deep freezer. A batch of 30 samples was subjected to RNA extraction using QIAamp viral RNA kits (Qiagen, Germany) as per the manufacturer’s instructions. Further, real-time reverse transcription-PCR (rRT-PCR) was performed using the RealStar SARS-CoV-2 RT-PCR kit v1.0, manufactured by Altona Diagnostics, Germany. A total of 11 RNA samples with a cycle threshold (*C_T_*) ranging from 17 to 21 were selected for whole-genome sequencing.

The NEBNext RNA Ultra II directional protocol was employed for total/viral RNA sequencing library preparation. The QIAseq DIRECT SARS-CoV-2 kit (Qiagen; catalog number 333898) was used for paired-end (PE) 2 × 150-bp sequencing on the Illumina HiSeq X sequencing platform (6). In total, 90% of the reads had a quality score above Q30. Based on the fastq file quality report, we trimmed the sequence reads to retain the high-quality sequences for further analysis. In total, 10 bases were trimmed from both the 3′ and 5′ ends using Cutadapt v1.15, and a minimum length of 32 bp was kept for downstream analysis. The preprocessed reads were then aligned to the human genome (hg19) using the STAR v2.4 aligner tool. The unaligned paired-end reads (left from the host transcriptome analysis) were aligned to the SARS-CoV-2 reference genome downloaded from NCBI RefSeq (accession number NC_045512.2). Alignment was performed using the BWA v0.7.12 aligner. The aligned reads were sorted, and then variant calling was performed using the LoFreq v2.1.2 variant caller and GATK. The variants identified were then annotated to the genes (7). The 11 consensus sequences generated using IGV and SAMtools, together with the SARS-CoV-2 reference genome (including the Wuhan strain), were used for multiple sequence alignment using CLUSTALW2 v2.1 software to inspect the nucleotide changes (8). The Nextstrain online browser and CoVsurver mutation app were used to assign the clades and identify the mutations, respectively. All tools were run with default parameters.

Demographic details of the hosts, NCBI GenBank and SRA identifiers, and sequencing metrics are provided in Table 1. Among the 11 sequenced samples, 9 were found to be Nextstrain clade 20B (PANGO lineage B.1.1.519, GISAID clade GR), and two were identified as clade 20A (PANGO lineage B.1.619, GISAID clade G). We found multiple single-nucleotide polymorphisms (SNPs) in each of the sequenced strains. The D614G mutation in the spike protein was observed in all the sequenced strains. Other identified amino acid changes are summarized in Table 1. Accumulating data from multiple laboratories will help us understand the SARS-CoV-2 genome better and aid human resource development and capacity building.

**TABLE 1:** Host demographic data, sequencing statistics, classification, and mutational analysis of 11 SARS-CoV-2 samples from asymptomatic hosts

Sample no.	BioSample ID (GenBank accession no.)	NCBI SRA accession no.	Host demographic data		Total data (Gb)	Avg % of reads >Q30	Avg base quality (%)	Avg sequencing depth (×)	Alignment to human genome (bp)	Alignment to SARS genome (bp)	Assembled SARS-CoV-2 genome size (bp)	Avg % GC	Nextstrain clade, GISAID clade, PANGO lineage	Amino acid changes
			Age	Gender^*a*^										
1	WGS-1/IND-BMCRI (MZ336026)	SRR14522713	19	F	2.76	91.0	35.6	81,741	9,119,198	17,426,966	29,899	38.0	20B, GR, B.1.1.519	S: D614G; ORF1a: S318L; ORF1b: P314L; ORF8: L4F; N: R203K, G204R
2	WGS-2/IND-BMCRI (MZ336027)	SRR14522712	36	M	2.83	90.7	35.5	85,143	9,371,311	17,957,149	29,899	37.9	20B, GR, B.1.1.519	S: D614G; ORF1a: S318L; ORF1b: P314L; N: R203K, G204R
3	WGS-3/IND-BMCRI (MZ336028)	SRR14522721	36	M	2.68	90.8	35.6	81,198	8,881,189	17,031,817	29,891	37.9	20B, GR, B.1.1.519	S: L18F, R102G, D614G; ORF1a: S318L, A3497V; ORF1b: P314L; N: R203K, G204R
4	WGS-4/IND-BMCRI (MZ336029)	SRR14522720	48	F	2.62	90.9	35.6	78,848	8,674,623	16,592,848	29,900	37.9	20B, GR, B.1.1.519	S: D614G; ORF1a: S318L; ORF1b: P314L; N: R203K, G204R
5	WGS-5/IND-BMCRI (MZ336030)	SRR14522719	27	F	2.9	91.1	35.6	87,077	9,609,139	18,446,073	29,901	37.9	20B, GR, B.1.1.519	S: D614G; ORF1a: S318L; ORF1b: P314L, G2180V; ORF3a: R6I; N: R203K, G204R
6	WGS-6/IND-BMCRI (MZ336031)	SRR14522718	58	M	2.69	90.9	35.6	80,471	8,905,879	17,021,528	29,901	37.9	20B, GR, B.1.1.519	S: D614G; ORF1a: S318L; ORF1b: M187I, P314L; ORF8: A51V; ORF9b: P10S; N: P13L, R195T, R203K, G204R
7	WGS-7/IND-BMCRI (MZ336032)	SRR14522717	23	M	2.67	90.9	35.6	80,559	8,852,338	16,962,841	29,887	37.9	20A, G, B.1.619	S: T76I, D614G; ORF1a: M3752I; ORF1b: P314L
8	WGS-9/IND-BMCRI (MZ336033)	SRR14522715	33	M	2.68	91.2	35.6	79,428	8,887,575	16,804,995	29,901	37.9	20A, G, B.1.619	S: D614G, D936Y; ORF1a: P2144S; ORF1b: P314L; ORF3a: Q57H, H243R
9	WGS-10/IND-BMCRI (MZ336023)	SRR14522714	58	M	2.81	90.9	35.6	82,914	9,303,222	17,588,163	29,895	37.9	20B, GR, B.1.1.519	S: D614G; ORF1a: D294Y, A1812D, K3577R; ORF1b: P255S, P314L, Y1759H; ORF3a: S58I, W131C, P240S; N: R203K, G204R
10	WGS-12/IND-BMCRI (MZ336024)	SRR14522710	20	M	3.25	92.2	35.8	97,850	10,766,698	20,723,042	29,888	37.9	20B, GR, B.1.1.519	S: D614G; ORF1a: P309S, D3042N; ORF1b: P314L; ORF3a: P104L; ORF8: C20F; M: A2V; N: R203K, G204R
11	WGS-13/IND-BMCRI (MZ336025)	SRR14522724	53	F	3.47	92.2	35.8	102,978	11,480,787	21,811,634	29,886	37.9	20B, GR, B.1.1.519	S: D614G; ORF1a: P1207L, H1838Y, D3042N, P3504H; ORF1b: P314L; ORF8: I121L; N: R203K, G204R

a M, male; F, female.

### Data availability.

These genome sequences have been deposited in the NCBI GenBank database under the accession numbers MZ336023, MZ336024, MZ336025, MZ336026, MZ336027, MZ336028, MZ336029, MZ336030, MZ336031, MZ336032, and MZ336033. The raw reads were also submitted to the NCBI SRA under the accession numbers SRR14522714, SRR14522710, SRR14522724, SRR14522713, SRR14522712, SRR14522721, SRR14522720, SRR14522719, SRR14522718, SRR14522717, and SRR14522715, respectively, under the BioProject accession number PRJNA728465.
